# Protective Role of High-Density Lipoprotein in Multiple Sclerosis

**DOI:** 10.3390/antiox13111276

**Published:** 2024-10-23

**Authors:** Agnieszka Damiza-Detmer, Małgorzata Pawełczyk, Andrzej Głąbiński

**Affiliations:** Department of Neurology and Stroke, Medical University of Lodz, ul. Zeromskiego 113, 90-549 Lodz, Polandandrzej.glabinski@umed.lodz.pl (A.G.)

**Keywords:** multiple sclerosis, HDL, antioxidant, anti-inflammatory, BBB, lipid alteration, apolipoprotein

## Abstract

Multiple sclerosis (MS) is a chronic, progressive demyelinating disease with a most likely autoimmune background and a neurodegenerative component. Besides the demyelinating process caused by autoreactive antibodies, an increased permeability in the blood–brain barrier (BBB) also plays a key role. Recently, there has been growing interest in assessing lipid profile alterations in patients with MS. As a result of myelin destruction, there is an increase in the level of cholesterol released from cells, which in turn causes disruptions in lipid metabolism homeostasis both in the central nervous system (CNS) and peripheral tissues. Currently, there is a growing body of evidence suggesting a protective role of HDL in MS through its effect on the BBB by decreasing its permeability. This follows from the impact of HDL on the endothelium and its anti-inflammatory effect, mostly by interacting with adhesion molecules like vascular cell adhesion molecule 1 (VCAM-1), intercellular adhesion molecule 1 (ICAM-1), and E-selectin. HDL, through its action via sphingosine-1-phosphate, exerts an inhibitory effect on leukocyte migration, and its antioxidant properties contribute to the improvement of the BBB function. In this review, we want to summarize these studies and focus on HDL as a mediator of the anti-inflammatory response in MS.

## 1. Introduction

HDL, along with LDL, is the main lipoprotein responsible for transporting cholesterol in the blood. Its primary function is to transport cholesterol from peripheral tissues to the liver. HDLs, similar to other lipoproteins—LDL, VLDL, IDL, are primarily made up of apolipoproteins (apo), non-structural proteins, and lipids. Their surface consists of a monolayer that includes amphipathic phospholipids, sphingolipids, and unesterified cholesterol, which encloses a core containing triglycerides and cholesteryl esters. HDLs differ in terms of size, density, and their specific lipid and protein makeup. The main apolipoprotein of HDL is apoA1 [1,2,3].

HDLs are known for their protective, antiatherogenic properties, particularly their ability to promote cholesterol removal from vascular cells and aid in its elimination. Beyond cholesterol efflux, they also have anti-inflammatory antioxidant effects and can stimulate nitric oxide (NO) production in the endothelium. Recent research has emphasized HDL functionality, revealing that its role in cardiovascular health may be more predictive than simply measuring HDL-C levels in the blood [2,4,5].

MS is a disorder that is of unwavering interest to researchers. Despite many years of study, the pathophysiology remains unclear. Yet, most investigators are convinced about the autoimmune nature of MS, with an emphasis on its neurodegenerative component. It is still unknown if the trigger for the cascade leading to the demyelinating process occurs within or outside the CNS [6]. Clinically, we can distinguish four subtypes of MS. In total, 80% of patients suffer from relapsing-remitting MS (RRMS). Patients have acute relapses followed by periods of remission. Unfortunately, for over 40% of patients, these remissions are partial, leading to disability over time. Untreated RRMS, or RRMS treated with insufficient response, can transform into secondary progressive MS (SPMS)—this occurs in 50–60% of RRMS cases. Primary-progressive MS (PPMS) is characterized by constant neurological worsening without acute relapses, and it affects about 10–15% of MS patients. The rarest subtype is progressive-relapsing MS (PRMS), where patients experience progressive worsening of the neurological state along with acute relapses [7].

Despite advances in understanding MS and treatments to reduce relapses, stopping and reversing disease progression remains a challenge. Current clinical approaches need to shift towards biologically based definitions of MS progression. Progression is driven by various mechanisms—non-resolving inflammation, neurodegeneration, oxidative stress, mitochondrial dysfunction, and failed compensatory responses like remyelination. These processes differ among patients and over time, highlighting the need for personalized tracking methods. New tools are needed to correlate clinical, radiological, and biological data, shifting from traditional MS classifications to a biomarker-driven, individualized treatment model. Transitioning to this new framework will require collaboration across research, healthcare, and regulatory systems [8].

In the autoimmune background of MS, autoreactive lymphocytes play a key role, particularly T CD4+ and CD8+ cells, especially CD4+ Th cells [9]. Th1 and especially Th17 seem to be the most important factors leading to the damage of endothelial cells (ECs) forming the BBB. B lymphocytes, which, similarly to T lymphocytes, are activated in the periphery or in the CNS, pass through the damaged BBB, and, as plasma cells, secrete autoantibodies. Myelin oligodendrocyte glycoprotein (MOG), myelin basic protein (MBP), myelin-associated glycoprotein (MAG), or myelin proteolipid protein (PLP) are postulated as antigens for these cells. Newer research indicates that the target for these produced antibodies may be lipid elements that build cell membranes, with the most likely antigen being cholesterol [10,11,12].

A crucial pathomechanism for MS is BBB damage, which leads to increased permeability and is associated with disease severity [13]. It is still unclear whether the initiating event of autoimmunity leading to BBB impairment occurs within the CNS or in peripheral tissues. It is also unknown whether the dysfunction of the endothelium that forms the BBB is primary, due to a pathological response to oxidative stress, or secondary, in response to previously circulating autoreactive lymphocytes [6,7]. Recent studies have shown that a damaged BBB exhibits excessive expression of P-, E-, and L-selectins, as well as VCAM and ICAM on ECs, initiated by exaggerated production of proinflammatory chemokines [14,15].

## 2. Methods

This narrative review draws upon a selection of research studies investigating the antioxidant and anti-inflammatory roles of HDLs in MS, particularly in relation to BBB dysfunction. Relevant original research, editorials, and review articles published up to August 2024 were sourced from the PubMed database. The search utilized keywords such as “HDLs”, “high-density lipoprotein”, “apoA-I”, “apoA1”, “multiple sclerosis”, “blood-brain barrier”, “endothelial dysfunction”, “antioxidative”, and “anti-inflammatory” to identify pertinent studies for inclusion in this review.

## 3. Lipid Profile Alteration and Their Association with MS

The CNS is the most lipid-rich organ. Approximately 50–60% of its dry weight is constituted by lipids, and 20–25% of that is cholesterol. Up to 80% of brain cholesterol is used in building myelin sheaths. In addition to cholesterol, myelin also contains other lipids, such as phospholipids and glycosphingolipids [16,17]. Cholesterol is synthesized de novo in the brain, mostly by astrocytes in the mature CNS and by oligodendrocytes and neurons in the postnatal period. Due to the fact that the BBB is impermeable to cholesterol, its metabolism—from synthesis to transport and storage—is almost completely independent of metabolism in the periphery [16,18]. Similarly to peripheral tissues, cholesterol in the brain is transported by lipoproteins. Due to the impermeability of the BBB to these molecules, the profile of lipoproteins in the CSF and serum is different. CSF is abundant in apolipoprotein E (apoE), apolipoprotein A1 (apoA1), and apolipoprotein J (apoJ), while apolipoprotein B (apoB) is absent. Analysis of the density and lipid composition of lipoproteins in CSF reveals that they most closely resemble HDL found in the periphery, hence their name HDL-like. However, HDL-like particles are larger than their peripheral counterparts [19,20]. Proteins from the ATP-binding cassette transporter (ABC) family—ABCA1, ABCG1, and ABCA7—are required during the formation of lipoproteins. These transporters are located in the neuronal cell membrane [19]. They are the main proteins involved in the transmembrane transport of cholesterol from the cell. They are responsible for the incorporation of phospholipids into apoE, resulting in the formation of a lipoprotein disk—nascent HDL, which subsequently incorporates free cholesterol produced in the cell. This cholesterol undergoes esterification by enzymes, most likely cholesterol ester transfer protein (CETP) and lecithin cholesterol acyltransferase (LCAT), resulting in the formation of mature HDL-like lipoproteins. The transport of cholesterol across the cell membrane using ABC transporters is energy-consuming, requiring the use of ATP [21,22,23]. Neurons, microglia, and arachnoid cells use these mature lipoproteins via low-density lipoprotein receptors (LDLR), mostly LDLR-related protein 1 (LRP1). Transport through these receptors is strongly associated with apoE [17]. In order to maintain cholesterol homeostasis, in addition to its synthesis and metabolism, the process of its removal from cells must take place. Cholesterol is metabolized to oxysterols or undergoes esterification. This process takes place thanks to oxidizing enzymes—sterol hydroxylases. Most of these enzymes belong to the cytochrome P450 complex. The main route of elimination of excess cholesterol is hydroxylation by the enzyme CYP46A1, resulting in the metabolite 24S-hydroxycholesterol (24S-OHC). Due to its hydrophilic nature, 24S-OHC is able to cross the BBB and is found in serum. Peripherally located 24S-OHC undergoes esterification, binding with fatty acids to form LDL or HDL [18,24,25]. Oxysterols, apart from their function as cholesterol transporters, are also regulators of their metabolism, among other roles, by acting as ligands for liver X receptors (LXRs). LXRs activate the transcription of genes, promoting cholesterol efflux from the cell, which causes a decrease in its intracellular level [18,26,27]. Increased levels of oxysterols, especially 24S-OH, are found in the serum of MS patients [28,29,30]. Because they freely migrate across the BBB, they have an activating effect on LXRs [28]. These receptors affect inflammatory responses via T lymphocytes. They reduce their influx into the CNS, inhibit the differentiation in naive CD4+ T lymphocytes into Th17-producing cells, and inhibit Il-9-producing CD8+ T lymphocytes [31]. In addition, LXR activation has an anti-inflammatory effect, which is achieved by inhibiting proinflammatory genes responsible for the production of TNFα, IL-1β, IL6, and nitric oxide synthase [32]. The remyelination process is also associated with the activation of LXRs. This is evidenced by studies on mice, in which blocking LXRs inhibits the repair processes within myelin while their activation stimulates the maturation of oligodendrocytes and both myelin production and remyelination. This mechanism involves reverse cholesterol transport using ABCA1 and apoE [33]. Studies also suggest LXRs’ role in reducing oxidative stress by stimulating antioxidant genes: glucose-6-phosphate dehydrogenase (G6PDH), isocitrate dehydrogenase 1 (IDH1), and 6-phosphogluconate dehydrogenase (6-PGDH) in damaged myelin sheaths in peripheral nerves [34]. Many studies provide evidence that MS is associated with serum lipid profile alteration. The most common findings in these patients are increased levels of TC, LDL, TG, and HDL [35,36,37,38]. However, it is not known whether the dyslipidemia described in the studies is the result of myelin damage associated with the development of the disease or if it is pathophysiologically unrelated to the processes underlying MS and is merely a comorbidity.

Many studies provide evidence that MS is associated with serum lipid profile alteration. The most common findings in these patients are increased levels of TC, LDL, TG, and HDL [24,35,39,40]. A recent study assessed the prevalence of normolipidemia and various types of dyslipidemia in a group of healthy individuals, those with RRMS, and those with progressive MS (PMS, which included patients with PPMS and SPMS). While it did not show differences in the occurrence of different types of dyslipidemia between the groups, it was observed that individuals with PMS and higher HDL levels demonstrated lower levels of disability [41]. There are also studies discussing the impact of diet on the course of MS. One study shows that patients following a diet high in saturated fats present greater disability and experience a more severe course of the disease [42]. Meanwhile, a more recent study—a randomized clinical trial—points to the Swank diet (low in saturated fats) and the Wahls diet (modified Paleolithic elimination) as diets that reduce chronic fatigue and improve quality of life [43]. However, it is not known whether the dyslipidemia described in the studies is the result of myelin damage associated with the development of the disease or if it is pathophysiologically unrelated to the processes underlying MS and is merely a comorbidity.

Various studies indicate a positive correlation between elevated levels of TC and the Expanded Disability Status Scale (EDSS), TC and disease duration, increased levels of LDL and EDSS, and LDL with disease duration. Moreover, levels of TC and LDL are significantly higher in PPMS and SPMS than in RRMS [44,45,46,47]. The relationship between high TC levels and a higher EDSS score may result from the impact of excess cholesterol on remyelination. Some studies suggest that the toxic effect of excess cholesterol in the CNS has an inhibitory effect on remyelination by limiting microglia’s ability to clear myelin debris [33]. Other studies have shown an association between increased levels of LDL and contrast-enhancing lesions (CEL), the number of lesions, or the appearance of new lesions in T2 MRI sequences [38,48,49]. Increased levels of TC and its metabolites—24S-OHC and 27S-OHC—are also found to positively correlate with CEL. Furthermore, they are negatively correlated with brain volume in MS patients [45,50].

Other studies have shown an association between increased levels of LDL and CELs, the number of lesions, or the appearance of new lesions in T2 MRI sequences [40,51,52]. Increased levels of TC and its metabolites—24S-OHC and 27S-OHC—are also found to positively correlate with CELs. Furthermore, they are negatively correlated with brain volume in MS patients [30,47].

The lipid metabolism disorder described above results from disturbances in cholesterol homeostasis in the brain. The destruction of myelin—the process that underlies MS—leads to an overload of the CNS with released cholesterol. This necessitates enhanced activity of the processes responsible for its removal in order to reduce the risk of its toxic effects [36]. The activity of CYP46A1 hydroxylase is increased, which metabolizes excess cholesterol to 24S-OHC, allowing it to freely penetrate the BBB. In serum, 24S-OHC is mainly transported in the form of LDL (70–80%) and HDL (20–30%) and then eliminated in the liver as bile acids [37]. Through the damaged BBB, cholesterol originating from the demyelination process penetrates into the bloodstream, which may indirectly result in increasing levels of serum LDL and TC.

Activation of LXR receptors takes place both in the CNS and in the periphery, mainly in the liver. Ligands for these receptors (including 24S-OHC) elicit an increase in the transcription of genes for ABCA1, ABCG1, and apoE, which are responsible for reverse cholesterol transport. This indirectly leads to a further increase in LDL, HDL, and VLDL levels in the serum. The increase in apoE levels is a response to the increased need for cholesterol efflux from the cell. Activation of LXRs also induces the promotion of transcription of the SREBP1c gene, leading to an increase in the synthesis of fatty acids. Another extremely important function of LXR is its immunomodulatory role. These receptors, located on T lymphocytes and macrophages, inhibit the influx of these cells into the CNS under conditions of stimulation. Activation of LXR also leads to a decrease in the transcription of genes responsible for the production of TNFα, IL-6, and IL-1β and also promotes transcription of anti-inflammatory factors such as IL-10 and TGFβ [31,32,38].

The increase in LDL levels resulting from the mechanisms described above is also harmful in itself. Under normal conditions, even increased LDL does not affect the processes occurring in the CNS due to the preserved integrity of the BBB. However, in the course of MS, when there is damage to the BBB and a significant increase in its permeability, even particles as large as LDL can migrate through it freely. In the vessel walls, oxidation occurs, leading to the formation of ox-LDL, which has a very strong atherogenic and proinflammatory effect. Its proinflammatory effect results from the presence of a changed epitope of apoB, the main apolipoprotein of LDL [53,54]. The pathologically changed epitope becomes recognizable by macrophages, which “engulf” ox-LDL, leading to the formation of foam cells, whose role in demyelination has been suggested [50,55]. In this process, chemotaxis of monocytes occurs, promoting the production of proinflammatory cytokines such as TNFα, IL-6, and IL-8 [48]. As a consequence of these modifications, HDL shifted from being an anti-inflammatory particle to a proinflammatory one, as evidenced by its altered capacity to either prevent or promote LDL oxidation by arterial wall cells or to either inhibit or enhance LDL-induced monocyte chemotactic activity [49]. All this exaggerates the inflammatory and demyelinating processes in the CNS. This is reflected in previously conducted studies showing a positive relationship between the concentration of LDL, ox-LDL, and the level of EDSS, the duration of the disease, or the number of demyelinating plaques in the CNS [53,56].

## 4. HDL’s Role in MS Pathophysiology

HDL is one of five classes of lipoproteins. This classification is based on molecular size, density, and the content of individual lipids and apolipoproteins. Both LDL and HDL are cholesterol-rich lipoproteins. The main apolipoprotein of HDL is apoA1 [3]. ApoA1 plays a crucial role in lipid metabolism. It facilitates the removal of fats from cells, including macrophages overloaded with oxidized LDL, by transporting them to other locations, such as the liver, for excretion. ApoA1 acts as a cofactor for LCAT, which forms plasma cholesteryl esters and may have an anticoagulant effect by stabilizing prostacyclin [57]. 

Research assessing HDL levels in patients with MS produces heterogeneous results. Most of the studies exhibit increased levels of HDL in MS. The adjustment of HDL varies among the different MS subtypes. The research conducted by Giubilei et al. assessed HDL levels in patients with clinically isolated syndrome (CIS). CIS is defined as the first episode of characteristic neurological symptoms that do not yet fulfill MS diagnostic criteria. In this study, patients with initially higher HDL levels also exhibited lower initial EDSS scores and a reduced number of CELs [51].

In the stable course of RRMS without recent relapses, HDL levels in serum are found to be significantly higher compared to healthy controls. Interestingly, the studies did not establish a significant correlation between EDSS scores, disease duration, and HDL levels [39,58,59]. 

Further, studies conducted by Meyers et al. and Gardner et al. showed reduced levels of HDL and apoA1—the main apolipoprotein for brain HDL, in the group of patients with MS. The results obtained by these researchers may be due to the fact that their RRMS patients were older and had more advanced diseases compared to the previous studies or had already been diagnosed with PPMS or SPMS [60,61].

In the study assessing the differences in lipid profiles in patients with RRMS and SPMS, it was shown that HDL levels are significantly lower in patients with SPMS, suggesting a potential association of this lipoprotein with disease progression and severity [46].

Differences observed in HDL levels, whether elevated or decreased, as seen in the aforementioned studies, may also depend on the assessment of specific HDL subfractions—such as small and large HDL particles—as well as the evaluation of oxidized HDL (ox-HDL).

Analyzing studies that evaluate HDL levels in relation to the pathophysiology of MS, it is hypothesized that elevated HDL levels are associated with better prognosis, slower disease progression, and improved BBB function. Initial support for this hypothesis comes from observed differences between the subtypes of multiple sclerosis.

Prospective studies evaluating patients with RRMS over a 5-year period have demonstrated that individuals with lower baseline HDL levels exhibit a significantly higher risk of developing SPMS [47]. Additionally, correlations have been observed between HDL levels and brain imaging findings on MRI. It has been demonstrated that during the course of the observations, patients with relative increases in HDL levels exhibited reduced gray matter atrophy [47,62]. Some available data indicate that higher levels of HDL and apoA1 are associated with improved cerebral perfusion in patients with MS [58]. 

Many studies also indicate a relationship between HDL and BBB integrity. An indirect marker of this correlation is the association between higher HDL levels and a reduced number of CELs, smaller lesion volumes, and a prospectively lower incidence of new plaque formation [39,40,58,63]. Fellows et al. designed a study to investigate the direct relationship between HDL and BBB integrity. They compared serum HDL levels with markers of BBB damage found in CSF. They discovered that higher levels of HDL and apoA1 are associated with lower total protein levels in CSF, lower albumin and IgG levels in CSF, and reduced concentrations of CD80+ and CD19+ cells [64]. These findings suggest that HDL is essential for maintaining proper BBB permeability. Similarly, the protective effects of HDL have been confirmed in Alzheimer’s disease, and studies have also demonstrated a relationship between higher HDL levels and reduced BBB permeability [65]. 

## 5. Mechanisms of HDL Action in MS

The exact mechanism underlying the role of HDL in the pathophysiology of MS is not fully understood. However, based on the studies mentioned, the most likely explanation is its beneficial effect on the BBB through its influence on the vascular endothelium. HDL exerts its effects on the endothelium through its anti-inflammatory, antioxidant, antithrombotic, antiapoptotic, and nitric oxide synthesis properties [2,3].

### 5.1. Anti-Inflammatory Effect of HDL

The anti-inflammatory properties exhibited by HDL result from various mediated processes. One of these processes is the protective effect of HDL on the vascular endothelium through its interaction with endothelial adhesion molecules such as VCAM-1, intercellular adhesion molecule-1 (ICAM-1), and E-selectin [66]. These molecules are involved in the processes of leukocyte rolling along the endothelium, their activation, adhesion, and subsequent transendothelial migration to the target site. In addition to facilitating leukocyte migration to sites of inflammation, the endothelium also participates in the production of cytokines and growth factors. These include IL-1, IL-6, IL-18, TNF, and monocyte chemotactic protein 1 (MCP-1) [15,67,68,69]. The overexpression of these molecules and the consequent damage to the BBB are well-documented in MS [70]. The protective effect of HDL on the vascular endothelium that constitutes the BBB is attributed to the fact that this apoA1-containing lipoprotein exerts an inhibitory effect on cytokine-induced EC activation, preventing the expression of adhesion molecules. Some in vitro studies suggest that the inhibitory effect of HDL on the expression of VCAM-1, ICAM-1, and E-selectin is associated with TNF-α. In these studies, endothelial cells were activated by the addition of TNF-α and then incubated with HDL. Depending on the specific experimental setup, the expression levels of various adhesion molecules were subsequently assessed. The levels of VCAM-1 and E-selectin were significantly reduced in the presence of HDL [71,72].

**Figure 1:**
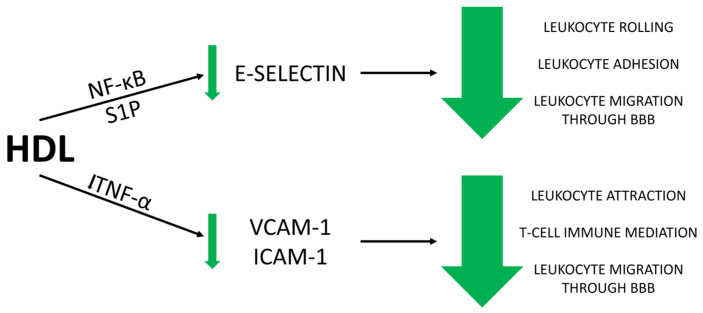
HDL reduces S1P levels by inhibiting sphingosine kinase, which in turn lowers NF-κB activity. This reduction in NF-κB activity leads to decreased expression of E-selectin, a molecule involved in leukocyte rolling, adhesion, and migration through the BBB. The downregulation of E-selectin by HDL results in a decrease in leukocyte rolling, adhesion, and migration through the BBB. HDL inhibits the effects of TNF-α, which is responsible for the upregulation of VCAM-1 and ICAM-1. By reducing the expression of VCAM-1 and ICAM-1, HDL diminishes leukocyte attraction and T-cell immune mediation, thereby reducing leukocyte migration through the BBB. BBB- blood–brain barrier, S1P—sphingosine-1-phosphate, NF-κB—nuclear factor kappa-light-chain-enhancer of activated B cells, HDL—high-density lipoprotein, TNF-α—tumor necrosis factor-alpha, VCAM-1—vascular cell adhesion molecule-1, ICAM-1—intercellular adhesion molecule-1.

Another mechanism by which HDL attenuates the endothelial activation response involves its modulation of E-selectin via nuclear factor kappa-light-chain-enhancer of activated B cells (NF-κB) and S1P [73,74]. HDL influences NF-κB by reducing S1P levels through the inhibition of the enzyme sphingosine kinase. Reduction in NF-κB levels leads to decreased expression of E-selectin [75,76]. While E-selectin contributes to leukocyte rolling, research indicates that its primary function is to facilitate the transition of leukocytes from rolling to firm adhesion to the endothelium. Experimental models lacking E- and P-selectin genes exhibit a complete absence of leukocyte migration across the BBB, an absence of leukocyte rolling, and increased leukocyte production [77]. Therefore, by reducing the expression of adhesion molecules, HDL reinforces the BBB, diminishes leukocyte migration into the CNS, and thereby reduces demyelination (Figure 1).

### 5.2. S1P Interactions with HDL

Changes in sphingolipid metabolism also play a significant role in the pathophysiology of MS. These changes involve not only the levels of sphingolipids themselves but also the signaling pathways mediated by them. Disruptions in sphingolipid metabolism are implicated in various neurodegenerative and inflammatory CNS diseases [78]. Sphingolipids participate in modulating the immune response, including through the induction of apoptosis in autoreactive T lymphocytes [79]. Sphingosine-1-phosphate (S1P) is a representative sphingolipid with a well-documented impact on immune response and neurodegeneration in MS [80]. S1P is involved in neuronal apoptosis and exhibits proinflammatory activity [80]. Increased expression of S1P receptors—primarily S1PR1, S1PR2, S1PR3, and S1PR5—has been observed in astrocytes and oligodendrocytes in MS patients [79,81].

**Figure 2:**
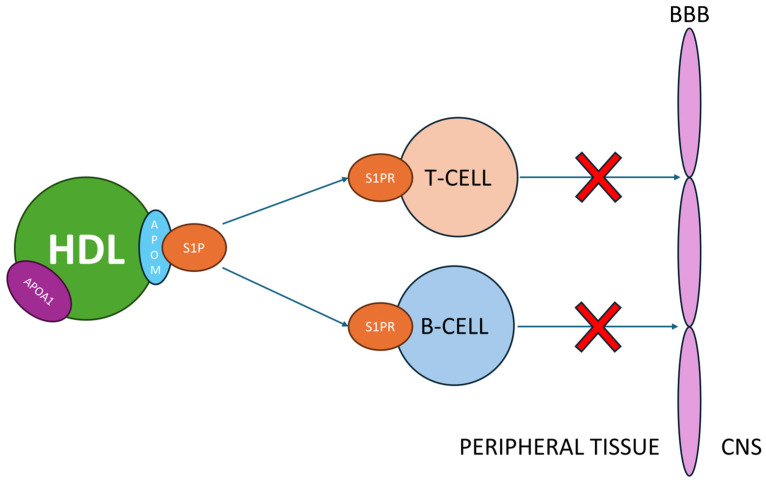
S1PRs (1–5) are abundantly expressed on immune cells, particularly on T and B lymphocytes. The primary function of these receptors is to regulate the movement of these cells between lymphoid organs, including lymph nodes, and peripheral tissues, such as the CNS. S1P molecules bound to HDL via apoM act as agonists for these receptors by binding them. Consequently, HDL indirectly influences immune response modulation and prevents activated T and B lymphocytes from migrating from lymphoid organs to the CNS, thereby inhibiting demyelination processes. HDL—high-density protein, CNS—central nervous system, BBB—blood–brain barrier, apoM- apolipoprotein M, S1PR—sphingosine-1-phosphate receptor, S1P—sphingosine-1-phosphate.

S1P is also modulated by HDL. Approximately 50–65% of serum S1P is bound to apolipoprotein M (apoM) and transported by HDL in the HDL-apoM-S1P complex. S1P receptors are responsible for various functions related to immune responses. Among them, S1PR1 is responsible for regulating the migration of leukocytes from secondary lymphoid organs to the bloodstream and lymph nodes [72,82,83,84]. One of the drugs used in the treatment of MS, fingolimod, is an agonist of S1P receptors. By binding to these receptors on autoreactive T and B lymphocytes, it prevents their migration into the CNS and retains them within lymphoid organs. In a study evaluating HDL levels in patients undergoing fingolimod therapy, it was demonstrated that HDL levels increased during treatment, which was associated with a deceleration of disease progression and a reduction in the frequency of relapses [85]. The results of this study suggest that the beneficial effect of HDL on the course and severity of MS is attributable to its interaction with S1P (Figure 2).

### 5.3. Antioxidant Effect of HDL

The role of oxidative stress in the development of MS is highly emphasized. The studies that were conducted support evidence of increased levels of oxidation and decreased activity of antioxidants in MS patients [86]. As mentioned before, in MS, we deal with the demyelination of neurons, axonal injury, and neurodegenerative components. These impairments could be associated with oxidative stress due to the high sensitivity of glial cells and neurons to its negative effects, such as DNA damage, mitochondrial dysfunction, or defective enzyme activities [86,87]. In patients with MS, elevated markers indicative of nitrosative stress are also observed, primarily including increased levels of nitrotyrosine [88,89].

**Figure 3:**
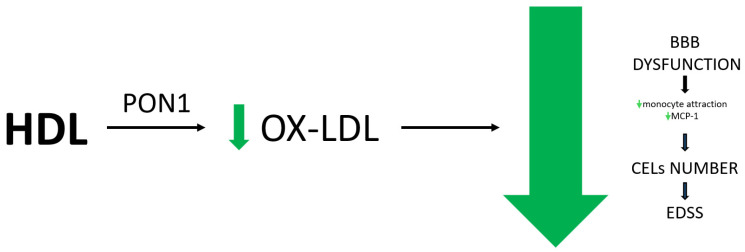
PON1 is an enzyme anchored in HDL. The activity of PON1 comprises the lactonase, homocysteine-thiolactonase (HTase), and arylesterase (AREase) activities. Through the hydrolysis of reactive compounds—PON1 is anticipating oxidation of LDL. The reduction in LDL oxidation to harmful proinflammatory ox-LDL in the course of MS leads to a decrease in the number of CELs. This is due to the fact that ox-LDL no longer exacerbates BBB endothelial dysfunction via increasing monocyte attraction or MCP-1 activation. As a result, there is a slowdown in the progression of disability, as measured by tools such as the EDSS.HDL—high-density lipoprotein, LDL—low-density lipoprotein, PON1—paraoxonase-1, BBB—blood–brain barrier, CELs—contrast-enhancing lesions, MCP-1—monocyte chemoattractant protein-1, EDSS—Expanded Disability Status Scale.

The antioxidant properties exhibited by HDL are also linked to their beneficial impact on the course of MS. These properties are attributed to HDL-associated enzymes, such as paraoxonase-1 (PON1) and glutathione selenoperoxidase (GSPx), whose dysfunction has been reported in the development of MS [90,91]. PON1 activity is found to be decreased in patients with MS compared to healthy individuals [92,93]. Moreover, patients with SPMS indicate lower PON1 activity compared to other subtypes [94]. The primary function of PON1 is to prevent the oxidation of LDL to ox-LDL, a process that occurs through the hydrolysis of the lactone ring in homocysteine thiolactone (HTL) molecules. Ox-LDL is known for its proinflammatory properties [95]. As previously mentioned, ox-LDL levels are elevated in patients with MS and are associated with higher EDSS scores and markers of endothelial dysfunction [53]. Those patients have also reduced levels of serum HDL. The relationship between HDL levels and ox-LDL has also been well-known for a long time, particularly in relation to atherosclerosis. Higher HDL levels reduce LDL oxidation, thereby exerting a protective effect on the vascular walls (Figure 3) [5]. 

Patients with MS who have higher HDL levels demonstrate better brain perfusion parameters [58]. This association may be attributed to the relationship between HDL and apoA1 with NO synthesis. Some studies suggest that HDL interacts with scavenger receptor-BI on brain vascular cells, stimulating endothelial NO synthase (eNOS) [96,97]. This enzyme is responsible for the synthesis of NO, which has vasodilatory properties. It has been shown that anti-ApoA1 antibodies can block eNOS-dependent vascular relaxation [97]. Another mechanism by which HDL exerts its vasodilatory effects on the vessel wall likely involves the mimicry of HDL-bound S1P3, which activates the Akt-mediated pathway leading to eNOS activation [98].

### 5.4. Ox-HDL in MS

The mechanisms outlined above highlight the potentially critical role of HDL in the pathogenesis of MS. However, several studies suggest that serum HDL levels may be diminished in patients with MS, particularly in those with more severe disease phenotypes such as PPMS, SPMS, or chronic RRMS [46,60,61]. This reduction may be linked to structural abnormalities in HDL, its oxidation to the proinflammatory ox-HDL, or potential dysfunctions in LXRs, which are pivotal in regulating cholesterol homeostasis within the CNS [99]. Ox-HDL In a study conducted by Jorissen et al., it was demonstrated that patients with RRMS with reduced HDL levels exhibit nitration and oxidation of tyrosine and tryptophan, along with a +126 amu modification on tyrosine residues in HDL3-derived apoA1 [99]. In these patients, the presence of ox-HDL was also observed, which correlates positively with higher EDSS scores [93]. The formation of this harmful molecule in MS patients is attributed to prolonged oxidative stress, nitrosative stress, and the dysregulated activity of heme oxygenase 1 (HO-1). HO-1 exerts a protective role against reactive oxygen species (ROS) and oxidative stress, and its upregulation mitigates the harmful impact of oxidized HDL. Several studies have reported increased activity of this enzyme in patients with MS [100,101,102,103,104].

## 6. Conclusions

1. HDL’s Role in MS Pathophysiology: HDL plays a potentially critical role in MS by influencing BBB integrity and vascular endothelium through its anti-inflammatory, antioxidant, and nitric oxide synthesis properties.
2. Mechanisms of HDL Action: HDL protects the endothelial cells forming the BBB by interacting with adhesion molecules like VCAM-1, ICAM-1, and E-selectin, thereby reducing leukocyte migration and BBB permeability. HDL also modulates endothelial activation through NF-κB and sphingosine-1-phosphate (S1P) pathways, enhancing its protective effect.
3. Impact of HDL on S1P and Nitric Oxide: HDL modulates S1P, a crucial molecule in MS pathogenesis, by binding it to apolipoprotein M (apoM). This interaction impacts leukocyte migration and is linked to therapeutic effects observed with fingolimod, an agonist drug that targets S1P receptors.
4. Antioxidant Properties: HDL’s antioxidant properties, linked to enzymes such as PON1 and GSPx, contribute to its protective role. Increased ox-HDL levels are associated with MS, potentially worsening disease outcomes. Due to the reduced PON1 activity, elevated ox-LDL levels are associated with increased disease severity, higher EDSS scores, and endothelial dysfunction
